# Implementation of antimicrobial stewardship programmes in private healthcare settings in Africa: A scoping review

**DOI:** 10.4102/hsag.v28i0.2104

**Published:** 2023-02-03

**Authors:** Andile P. Dlungele, Lehlohonolo J. Mathibe

**Affiliations:** 1 Division of Pharmacology (Therapeutics), Faculty of Health Sciences, University of KwaZulu-Natal, Durban, South Africa

**Keywords:** implementation, antimicrobial, stewardship, antibiotics, private, healthcare, Africa

## Abstract

**Background:**

An Antimicrobial Stewardship Programme (ASP) is one of the strategic objectives of the World Health Organization’s (WHO) global action plan to combat antimicrobial resistance. There have been numerous publications on the implementation of ASPs in both private and public sectors globally. However, there are no reviews and interpretive scholarly research publications on successful implementation of ASPs in private healthcare settings in Africa.

**Aim:**

The aim of this study was to systematically gather relevant information from published findings and to interpret those findings into a coherent body of lessons learnt from successful ASP implemented in private healthcare settings in Africa.

**Method:**

Google Scholar and PubMed, which are online databases, were extensively searched, and studies, which met the inclusion criteria for this review, were retrieved. A data-charting list was developed to extract relevant data.

**Results:**

Only six South African studies reported on successful implementation of ASPs in private healthcare settings in Africa. The main focus areas include locally driven prescription audits as well as pharmacist-led interventions.

**Conclusion:**

Although private healthcare settings in Africa utilise antibiotic therapy for various infectious diseases, reports on implementation of ASPs in these settings are limited. To win the battle against antimicrobial resistance, private healthcare settings in Africa need to implement evidence-based guidelines and report on the rational use of antibiotics.

**Contribution:**

The private healthcare sector in Africa needs to play a more meaningful role in the implementation of ASPs.

## Introduction

Antimicrobial resistance (AMR) is a global public health concern (Ajuebor et al. 2019:791). If ignored, there is a potential to reverse significant progress achieved in medicine and healthcare since the discovery of penicillin in the 1920s. This healthcare threat continues to increase among microbial clinical isolates such as *Escherichia coli,* when compared to non-clinical strains (Hung et al. 2019). The prevalence of resistance to antibiotics reported for *E. coli* ranged from 81.8% to 100% (Hung et al. 2019). Lower prevalence rates ranging from 17.7% to 53% have also been reported recently (Havenga et al. 2019:4396; Mesa-Varona et al. 2021:8). It is evident that irrational prescribing and indiscriminate use of antibiotics play a major role in the spread of AMR. This poses a major clinical and public challenge for everyone. There is thus a need for rigorous implementation of evidence-based antimicrobial management strategies in both public and private healthcare settings in Africa and globally (Abbo & Hooton 2014:175; NDoH 2019:2).

Antimicrobial stewardship programmes (ASPs) are those interventions that improve appropriate use and rational prescribing of antimicrobials (Majumder et al. 2020:4728). Briefly, ASP is concerned with careful and responsible use of antimicrobials. Selecting appropriate drugs for appropriate patients is an integrated and multidisciplinary approach. These programmes are aimed at optimising antimicrobial therapy for individuals, preventing misuse of antimicrobials and minimising collateral damage (Parker & Mattick 2016:432). Antimicrobial stewardship programmes also encourage consistent surveillance with regard to the implementation of prescribing guidelines and the provision of continuing medical education on utilisation of antibiotics (World Health Organization [WHO] 2019:2).

Recently, Brinks et al. (2016) reported that ASP interventions resulted in a significant reduction in average utilisation of antibiotics – from 101.38 (95% CI 93.05–109.72) defined daily dose (DDD) to 83.04 (74.87–91.22) DDD per 100 patient days (Brink et al. 2016:1022–1023). Also, a well implemented ASP may improve compliance regarding correct antibiotic choices, doses, administration times and duration (Brink et al. 2017:1230–1231). Boyles et al. (2017) found that the introduction of a dedicated prescription chart and multidisciplinary antibiotic ward rounds at Groote Schuur Hospital decreased antibiotic consumption and pharmacy costs for more than four successive years (Boyles et al. 2017:117). A meta-analysis of studies evaluating the effect of ASPs in inpatient settings in the USA showed that there was decreased antimicrobial use and an improvement in AMR patterns following the implementation of ASPs (Wagner et al. 2014:1211). A significant reduction in healthcare costs, especially relating to the direct cost of antimicrobials, and indirect costs such as reduction in hospital stay and improved therapeutic outcomes, have been recorded with ASPs in studies conducted in Saudi Arabia, Sweden and China (Amer et al. 2013:549–550; Hou et al. 2014:3; Lanbeck, Tennvall & Resman 2016:5). A global survey of stewardship activities revealed that only 14% of respondents in Africa and 53% in Asia had any form of ASP in place (Howard et al. 2015:3).

There have been numerous publications on the implementation of ASPs in public healthcare settings globally. However, there are no reviews and interpretive scholarly research publications on successful implementation of ASPs in private healthcare settings in Africa. Therefore, the aim of this study was to systematically gather relevant information from published findings and to interpret those findings into a coherent body of lessons learnt from successful ASP implemented programmes in private healthcare settings in Africa. In particular, this article seeks to answer the following main questions: (1) Are there measures in place in private healthcare settings in Africa for the prevention of AMR? (2) How successful are ASPs in the private healthcare sector in Africa?

## Methods

The scoping review was informed by Arksey and O’Malley (2005:3–15). Briefly, this approach allows for objective, systematic and inclusive identification and screening of relevant literature. Data were searched from Google Scholar and PubMed databases on 25 June 2022. The keywords that were entered into the search engine, either on their own or in combination to create Boolean phrases, were implementation, antimicrobial, stewardship, antibiotics, private, healthcare and Africa.

### Inclusion and exclusion criteria

Studies that reported on interventions such as audits and feedback or reports, education or training programmes, use of treatment guidelines and formularies to improve antimicrobial prescribing and utilisation in private healthcare settings in Africa were included. Only reports and studies that were published by 25 June 2022 were included.

Reports on ASP implementation outside of African private healthcare were excluded. Surveys and opinion pieces on antibiotic consumption reports, which were not based on primary research, were also excluded, as were studies published in languages other than English.

### Reliability and validity of data extraction and processing

**FIGURE 1:**
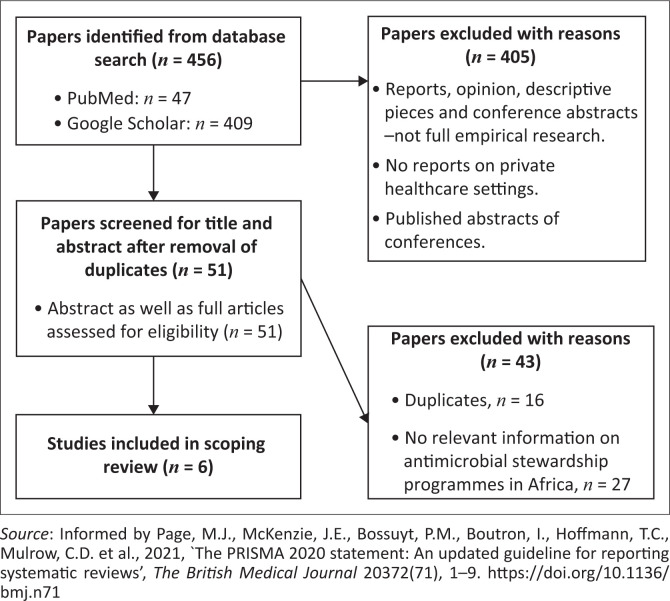
PRISMA flow diagram of the review process.

A data charting list was developed and used to extract the relevant data for this scoping review. Data regarding the year of publication, study design, country of origin, intervention type, team composition, study duration and outcomes were extracted. After each stage, the two authors met to discuss the results of their independent searches. Any emerging issues or discrepancies were resolved through consensus. This enhanced reliability, validity and reproducibility of the findings. Figure 1 illustrates the review process.

### Ethical considerations

Ethics approval was not required for this study as the data are freely available in the public domain. This article followed all ethical standards for research without direct contact with human or animal subjects.

## Results

As depicted in Figure 1, 456 studies were identified from dataset search. However, only six (*n* = 6) articles, which were conducted in South Africa and published after 2012, met the inclusion criteria for this scoping review.

### Main focus areas

Main focus areas of the six studies included in this scoping review were (1) ASPs and (2) prescription audits and pharmacist-led interventions.

#### Antimicrobial stewardship programmes

Chunnilall et al. (2015) evaluated antibiotic prescriptions and assessed the indications, choice of antibiotic, dosage and duration of therapy. Adherence to South African treatment guidelines (national standard treatment guidelines [STGs]) and the Essential Medicines List (EML) was investigated (Chunnilall et al. 2015:18). They reported a 45% and 69% adherence to treatment guidelines and recording of diagnoses, respectively (with or without the international classification of diseases, i.e. ICD10 codes). Also, Chunnilall et al. (2015) found that 26% of patients did not require an antibiotic, 13% were given an incorrect dose and 12% were given an incorrect drug (Chunnilall et al. 2015:21). In summary, Chunnilall et al. (2015) showed that, largely, there were inappropriate antimicrobial prescribing practices with regard to incorrect doses, duration of administration of drugs, excessive reliance on empirical treatment strategies and poor adherence to treatment guidelines in private healthcare settings.

Van Den Bergh et al. (2020) assessed adherence to the South African community-acquired pneumonia (CAP) guideline in a multicentre prospective cohort study. The study consisted of four phases. Phase 1 dealt with the establishment of leadership structures and protocol development, phase 2 focused on baseline compliance, phase 3 was about intervention and phase 4, post intervention (Van Den Bergh et al. 2020:3–4). Post-intervention or implementation, overall CAP compliance improved from 47.8% to 53.6% (*p* < 0.0001), diagnostic stewardship compliance improved from 49.1% to 54.6%, and compliance with ASP measures improved from 45.3% to 51.6% (CI 4.0–8.6, *p* < 0.0001) (Van Den Bergh et al. 2020:6). They demonstrated that implementation and adherence to ASPs in private healthcare settings is feasible.

Messina et al. (2018) focused on the use of a specific antibiotic, evaluating the utilisation of Colistin in four private sector hospitals and the opportunities to improve its appropriate use in future (Messina et al. 2018:29). They reported a 99.0% compliance with obtaining a culture prior to antibiotic therapy, 93.5% compliance with prescription of a loading dose and 98.5% compliance regarding prescription of Colistin in combination with another agent. The overall composite compliance with the six Colistin stewardship process measures was 82.0% (Messina et al. 2018:29). The study identified multiple stewardship opportunities to optimise Colistin therapy in hospitalised patients.

#### Prescription audits and pharmacist-led interventions

In conjunction with audits analysing antimicrobial use, some institutions are in the process of implementing or have implemented successful ASPs (Brink et al. 2016). Studies were carried out across multiple sites within a hospital network. The three pharmacist-led studies or initiatives were carried out at Netcare hospitals (a private hospital group in South Africa) (Brink et al. 2016:1, 2017:1228; Messina, Van Den Bergh & Goff 2015:S5). In particular, Messina et al. (2015) carried out a prospective multi-centre study across 33 hospitals to evaluate hang-time compliance of the initiation of first doses following new antibiotic orders. A guide was created to implement the different stages of the project (Messina et al. 2015:S6). Briefly;

- ‘a hang-time educational poster was designed,
- this was followed by a 4-week implementation period that covered four stages:
- stage 1 looked at the initial hang-time compliance for a chosen ward (baseline);
- stage 2 consisted of pharmacist-led group education and training sessions, which included nurses;
- stage 3 included pharmacist-led monitoring and data collection of hang-time compliance using a standard template;
- and stage 4 involved submission of results to a study coordinator who tracked and collated results in real time with feedback’ (Messina et al. 2015:S7–S8).

This initiative led to a reduction in hang times with 78% of patients receiving the first antibiotic dose within 1 h of placing the order to fill prescriptions (Messina et al. 2015:S9).

Another pharmacist-led study aimed to measure adherence to peri-operative antibiotic prophylaxis (PAP) guidelines (Brink et al. 2017). This was a prospective audit with feedback for the development of an improved model for PAP. During the pre-implementation phase, the PAP guideline and process measures were tested and refined at pilot sites and then rolled out during the post-implementation phase to 34 Netcare hospitals (Brink et al. 2017:1228). In that study, the three process measures to determine adherence were as follows: (1) antibiotic choice, (2) dosage and (3) duration for which the medicine should be administered. The study also included an educational component, which was carried out via institutional workshops. This initiative showed improved compliance to process measures (Brink et al. 2017:1228–1229, 1231).

In 2016, Brink et al. looked at implementation of a pharmacist-driven ASP, and this project was run across 47 Netcare hospitals (Brink et al. 2016:1). The pre-implementation phase observed baseline ASP interventions, followed by a stepwise implementation process at each hospital, which included interventions to reduce antibiotic consumption. The process measures used were prolonged duration, multiple antibiotics and redundant antibiotic cover (Brink et al. 2016:3). The model was established, and then the post-implementation phase assessed antibiotic consumption via DDDs. The change in antibiotic consumption at each hospital required a pharmacist intervention (Brink et al. 2016:1). There was also a reduction in mean antibiotic DDDs from 101.38 (95% CI 93.05–109.72) to 83.04 (95% CI 74.87–91.22), *p* < 0.0001 (Brink et al. 2016:6–7).

## Discussion

Positive effects of antimicrobial stewardship interventions in low- and middle-income countries have been previously reported (Cox et al. 2017:816–817; Van Dijck, Vlieghe & Cox 2018:276). The outcome of this review, which focused on identifying a coherent body of lessons learnt from successful ASP implemented programmes in private healthcare in Africa, identified six primary studies. These findings suggest that most countries are yet to align with the global efforts to combat increasing antibiotic resistance through stewardship of available antibiotics.

The primary goals of ASPs are to improve clinical outcomes and reduce antibiotic resistance (Majumder et al. 2020:4728). All included studies used process measures to assess the effectiveness of interventions and the antimicrobial stewardship strategies or interventions employed in the included studies involved those that have been shown to improve antibiotic prescribing or use (Chunnilall et al. 2015:21; Messina et al. 2018:29). Interventions to improve hospital antibiotic prescribing are grouped as follows: (1) restrictive (formulary restriction), (2) audit and feedback with intervention, education and guidelines (Brink et al. 2016:1, 2017:1228; Chunnilall et al. 2015:21; Messina et al. 2015:S5; Van Den Bergh et al. 2020:6).

Successful antimicrobial stewardship requires a strong commitment from institutions’ senior management leadership to implement core elements of this programme (Pollack & Srinivasan 2014:597–598). Consequently, guidelines for institutional ASP recommend the establishment of a multidisciplinary team or committee with the responsibility of ensuring prudent antibiotic use. This committee should include an infectious diseases specialist or microbiologist and a pharmacist with infectious diseases training as core members (Majumder et al. 2020:4716). Among the included studies, only one described the involvement of a multidisciplinary team in the reported stewardship interventions (Van Den Bergh et al. 2020:2). The study identified the lack of sufficiently-trained personnel as a major barrier to successful ASP implementation. Pharmacists-led interventions, however, demonstrated that antimicrobial stewardship can be implemented in private healthcare settings.

### Limitations

This study presents with certain limitations. Only papers published in English were considered for this scoping review. There may therefore have been papers published in French, Portuguese, Spanish or other official languages in Africa that have reported on ASP programmes in private healthcare settings that were not included. It is, however, unlikely that this limitation affected the interpretation or conclusions of this study. The articles included in the present study highlighted the fact that studies on antibiotic stewardship in low- and middle-income countries are very limited.

### Recommendations

There is a dire need for empirical research on antimicrobial utilisation and implementation of measures to improve prudent prescribing and antibiotic stewardship in private healthcare settings in Africa. Individual institutions in private healthcare settings often function independently. However, to prevent AMR, there is a need for a legislative framework to compel these institutions to establish and report on ASPs. This is the responsibility of all healthcare providers regardless of the settings in which they operate.

## Conclusion

Antimicrobial therapy is the cornerstone of many infections managed in private healthcare settings and accounts for substantial utilisation of antibiotics (Bitterman et al. 2016:561.e13). However, the findings of this review suggest that ASPs are very limited and only scarcely-implemented in the private healthcare settings in Africa. This problem therefore requires urgent attention.
